# BCG-derived acellular membrane vesicles elicit antimycobacterial immunity and innate immune memory

**DOI:** 10.3389/fimmu.2025.1534615

**Published:** 2025-03-12

**Authors:** Takehiro Yamaguchi, Noriaki Samukawa, Sohkichi Matsumoto, Masayuki Shiota, Masaki Matsumoto, Ryoma Nakao, Satoru Hirayama, Yutaka Yoshida, Akihito Nishiyama, Yuriko Ozeki, Shuhei Tomita

**Affiliations:** ^1^ Department of Pharmacology, Osaka Metropolitan University Graduate School of Medicine, Osaka, Japan; ^2^ Department of Bacteriology I, National Institute of Infectious Diseases, Tokyo, Japan; ^3^ Department of Bacteriology, Niigata University Graduate School of Medical and Dental Sciences, Niigata, Japan; ^4^ Department of Bacteriology, Osaka Metropolitan University Graduate School of Medicine, Osaka, Japan; ^5^ Division of Research Aids, Hokkaido University Institute for Vaccine Research & Development, Sapporo, Japan; ^6^ Laboratory of Tuberculosis, Institute of Tropical Disease, Universitas Airlangga, Surabaya, Indonesia; ^7^ Department of Molecular Biology of Medicine, Osaka Metropolitan University Graduate School of Medicine, Osaka, Japan; ^8^ Department of Omics and Systems Biology, Niigata University Graduate School of Medical and Dental Sciences, Niigata, Japan; ^9^ Division of Microbiology and Infectious Diseases, Niigata University Graduate School of Medical and Dental Sciences, Niigata, Japan

**Keywords:** BCG, tuberculosis, acellular vaccines, membrane vesicles (MVs), trained immunity

## Abstract

Tuberculosis (TB) is one of the leading causes of death due to infectious disease. The sole established vaccine against TB is the *Mycobacterium bovis* Bacillus Calmette–Guerin (BCG) vaccine. However, owing to the lack of durable immunity with the BCG vaccine and its risk of infection, safer vaccines that can also be used as boosters are needed. Here, we examined whether membrane vesicles (MVs) from BCG (BCG-MVs) isolated from BCG statically cultured in nutrient-restricted Sauton’s medium (s-MVs) and from BCG planktonically cultured in nutrient-rich medium commonly used in the laboratory (p-MVs) could be used as novel TB vaccines. MVs are extracellular vesicles produced by various bacteria, including mycobacteria. Differences in the culture conditions affected the morphology, contents, immunostimulatory activity and immunogenicity of BCG-MVs. s-MVs presented greater immunostimulatory activity than p-MVs via the induction of TLR2 signaling. Mouse immunization experiments revealed that s-MVs, but not p-MVs, induced mycobacterial humoral and mucosal immunity, especially when administered in combination with adjuvants. In a BCG challenge experiment using BCG Tokyo type I carrying pMV361-Km, subcutaneous vaccination with s-MVs reduced the bacterial burden in the mouse lung to a level similar to that after intradermal vaccination with live BCG. Furthermore, the administration of s-MVs induced a significant lipopolysaccharide-induced proinflammatory response in macrophages *in vitro*. These results indicate that BCG-MVs obtained from static culture in Sauton’s medium induce not only humoral immunity against mycobacteria but also trained immunity, which can allow the clearance of infectious agents other than mycobacteria. Together, these findings highlight the immunological properties of BCG-MVs and the availability of acellular TB vaccines that confer broad protection against various infectious diseases.

## 1 Introduction

Tuberculosis (TB), caused by *Mycobacterium tuberculosis* (Mtb), is one of the oldest infectious diseases in human history and remains a leading cause of death worldwide. In 2022, 1.3 million people died from TB (1). The administration of a vaccine derived from *Mycobacterium bovis* bacillus Calmette–Guerin (BCG) is the sole established vaccination strategy against TB (1). The BCG vaccine prevents TB occurrence and severe forms of TB infection, such as meningitis and miliary disseminated TB, in infants (2, 3). On the other hand, the efficacy of the BCG vaccine against TB in adults is limited (3). Thus, there is a need to develop a more effective vaccine against TB for adult use. Recent studies have shown that intravenous administration of the BCG vaccine confers much stronger protection against Mtb infection than does conventional intradermal administration (4, 5). However, it remains unclear whether intravenous BCG vaccination confers protection against TB in all age groups or if it is applicable for human use. BCG is a live vaccine that sometimes causes adverse events, such as local infection in the bone, joints or skin and disseminated infection (6). To avoid such adverse events, a safer BCG or more effective acellular vaccine needs to be developed.

Bacterial membrane vesicles (MVs) are exosome-like nanoscale extracellular vesicles produced by bacteria. MVs contain various molecules derived from their donor bacteria, such as proteins, nucleic acids and lipids. Recently, MVs have been considered novel vaccine candidates because of their immunostimulatory properties (7–10). A previous study revealed that mycobacterial MVs from Mtb have greater adjuvanticity and immunogenicity than MVs from BCG (BCG-MVs) do (11). This difference in vaccine efficacy between Mtb-derived MVs and BCG-MVs may be caused by the presence of Mtb-specific antigens, such as CFP-10 and ESAT-6, in the Mtb-derived MVs (12, 13). However, vaccination with CFP-10- or ESAT-6-containing MVs may interfere with the latent TB diagnostic test and make distinguishing latent TB from controlled TB difficult (14, 15). Moreover, with respect to the biosafety of and ease of handling MV-donor bacteria, BCG may be more favorable than Mtb.

Owing to their exosome-like properties, MVs may serve as carriers that mediate intercellular communication among bacteria and between bacteria and the host (9). MVs as vaccines induce both adaptive immunity and innate immune memory, also known as trained immunity (16). For example, recent studies have shown that the BCG vaccine induces not only TB-specific immunity but also trained immunity (17, 18). BCG-induced trained immunity results in hypersensitive innate immune response against nonspecific pathogen-associated molecular patterns, including those derived from BCG and Mtb (19, 20). The underlying mechanism of this trained immunity is that BCG vaccination induces epigenetic alterations in the hematopoietic stem and progenitor cell (HSPC) compartment in the bone marrow and functional alterations in HSPCs. However, it is unclear why BCG vaccination can train HSPCs despite being administered at sites distant from the bone marrow. Thus, exosome-like BCG-MVs may be involved in training HSPCs.

In the present study, we investigated whether BCG-MVs could be used as novel TB vaccines by performing immunization experiments in mice. After generating two different BCG-MV preparations, s-MVs prepared from static-cultured BCG in nutrient-restricted Sauton’s medium, which is used to produce the BCG vaccine, and p-MVs prepared from planktonic-cultured BCG in nutrient-rich medium, which is commonly used in mycobacterial experiments, we found that s-MVs induced mycobacterial humoral immunity and reduced the bacterial burden in mice. In addition, we showed that s-MVs also induced trained immunity. In this study, we demonstrated the possibility of developing novel TB vaccines using BCG-MVs from static culture in Sauton’s medium, which may also protect against other infectious diseases.

## 2 Materials and methods

### 2.1 Bacterial strains and culture conditions

We used BCG Tokyo type I, an attenuated strain of *M. bovis*, in this study. The frozen BCG stock was thawed and cultured in Middlebrook 7H9 broth (BD, Franklin Lakes, NJ, USA) supplemented with 0.2% (v/v) glycerol, 0.5% bovine serum albumin, 0.081% NaCl, 0.2% d-glucose, and 0.05% (v/v) Tween 80 (7H9ADN) (21). The morphology of BCG was assessed by scanning electron microscopy (SEM) (22).

To isolate static-cultured MVs, when the turbidity of the bacterial suspension (OD600) reached approximately 1.0, 10 mL of the BCG culture was added to 200 mL of Sauton’s medium (4.0 g/L L-asparagine monohydrate, 2.8 g/L Trisodium citrate dihydrate, 0.5 g/L K_2_HPO_4_, 0.5 g/L MgSO_4_·7H_2_O, 0.05 g/L ammonium iron citrate, and 6% glycerol), which is commonly used to produce the BCG vaccine. BCG was then statically cultured for 21 days. To isolate the planktonic-cultured MVs, 10 mL of the BCG culture was added to Mueller−Hinton II (MH-II) broth (BD) supplemented with 0.05% (v/v) Tween 80 or 7H9ADC broth, which is commonly used for mycobacterial experiments. Then, BCG was planktonically cultured for 10–14 days with gentle stirring by the magnetic string bar at 60 rpm.

For the mycobacterial challenge experiment, we used the pMV361-Km-containing BCG mutant strain (23, 24), which was kindly provided by Dr. Yoshitaka Tateishi, Niigata University. Kanamycin (Km) was purchased from FUJIFILM Wako Pure Chemical Corporation (Osaka, Japan).

### 2.2 MV isolation

BCG-MVs were isolated via ultracentrifugation according to previous methods (11, 13, 25). Briefly, the culture supernatant was harvested via centrifugation (10,000×g for 30 min at 4°C) and passed through 0.45 μm and 0.22 μm filters. The supernatant was subsequently concentrated via ultrafiltration using an Amicon stirred cell and a 100 kDa Ultracel membrane (Millipore, Burlington, MA, USA). The MVs were sedimented via ultracentrifugation (100,000×g for 2 h at 4°C), washed once with phosphate-buffered saline (PBS) and resuspended in PBS. The BCG-MV protein concentration was determined via a BCA assay (FUJIFILM Wako) according to the manufacturer’s instructions. MVs from *Escherichia coli* Nissle 1917 *ΔflhD* (EcN-MVs), a probiotic strain lacking flagella, were purified from the supernatant of a 16-h bacterial culture of EcN after glycine induction performed as described previously (26). The EcN-MV protein concentration was determined via a Bradford assay (Bio-Rad Laboratories, Hercules, CA, USA). The morphology of the MVs was assessed by transmission electron microscopy (TEM) and field emission SEM (FE-SEM) (7). The size distributions of the MVs were assessed using a nanoparticle size analyzer NanoFCM (NanoFCM Inc., Xiamen, China) (7).

### 2.3 Macrophage culture

Human monocytic leukemia (THP-1) cells, which were kindly provided by Dr. Mayuko Osada-Oka, Kyoto Prefectural University, were cultured in RPMI-1640 medium (FUJIFILM Wako, Osaka, Japan) supplemented with 10% fetal bovine serum (FBS), and 100 units/mL penicillin plus 100 µg/mL streptomycin (P/S) in a humidified atmosphere of 5% CO_2_ at 37°C. THP-1 cells were seeded onto 12- or 24-well plates at densities of 5×10^5^ or 2.5×10^5^ cells per well, respectively. The cells were differentiated into Mφs with 100 nM phorbol 12-myristate-13-acetate (Cayman Chemical, Ann Arbor, MI, USA) in Dulbecco’s modified Eagle’s medium (DMEM) (FUJIFILM Wako) supplemented with 10% FBS and P/S for 24 h (27, 28). Then, the cells were washed twice with PBS, and the culture medium was replaced with fresh DMEM containing 10% FBS and P/S for 24 h. The cells were subsequently stimulated with BCG-MVs for 3 h (for the RNA experiment) or 24 h (for the multiplex assay).

### 2.4 Real-time qPCR

Total RNA was extracted with Isogen (Nippon Gene, Toyama, Japan) and a Direct-zol™ RNA MicroPrep kit (Zymo Research, Irvine, CA) as previously reported (29). After the quality of the RNA was checked with a NanoDrop 2000 (Thermo Scientific, Waltham, MA, USA), cDNA was synthesized using ReverTra Ace qPCR RT Master Mix with gDNA Remover (Toyobo, Osaka, Japan). Gene expression levels were quantified using Luna Universal qPCR Master Mix (New England BioLabs Inc., Ipswich, MA, USA). The sequences of the primer sets used in this study are listed in **Table 1**. PCR was performed on a 7500 Fast instrument (Applied Biosystems, Carlsbad, CA, USA). To normalize the data, the relative expression levels of the target genes were calculated via the comparative CT (ΔΔCT) method and compared with those of 18S rRNA as an internal standard. The experiments were performed independently three times.

**Table 1:** List of qPCR primer used in this study.

Oligonucleotides used for qPCR		
Target gene	Forward primer	Reverse primer
18S rRNA	GCAATTATTCCCCATGAACG	GGGACTTAATCAACGCAAGC
*Il-1b*	ATGATGGCTTATTACAGTGGCAA	GTCGGAGATTCGTAGCTGGA
*Il-6*	ACTCACCTCTTCAGAACGAATTG	CCATCTTTGGAAGGTTCAGGTTG
*Il-10*	GACTTTAAGGGTTACCTGGGTTG	TCACATGCGCCTTGATGTCTG
*Mcp1*	AGTCTCTGCCGCCCTTCT	GTGACTGGGGCATTGATTG
*Tnfa*	GAGGCCAAGCCCTGGTATG	CGGGCCGATTGATCTCAGC

IL, interleukin; MCP1, monocyte chemotactic protein 1; TNFα, tumor necrosis factor alpha.

### 2.5 Multiplex assay

The culture supernatant was collected from THP-1 Mφs stimulated or not by BCG-MVs. The concentrations of cytokines and chemokines in the supernatants were assessed via the Bio-Plex Pro™ Human Cytokine 17-Plex assay (Bio-Rad Laboratories) according to the manufacturer’s instructions. The fluorescence intensity was measured and the concentrations of the target factors were calculated with a Bio-Plex 200 system on the basis of Luminex multiple analyte profiling technology.

### 2.6 Animals and immunization

All animal procedures were approved by the animal experiment committee of Osaka City University (approval no. 18077) and were performed in accordance with our institutional animal care guidelines. Female BALB/c mice aged 6–8 weeks at the time of immunization were purchased from Japan SLC (Hamamatsu, Japan). The mice were randomly divided into cages according to their group, provided *ad libitum* access to food and water, and maintained on a 12-h light/dark cycle at 22 ± 1°C. The mice were intranasally or subcutaneously immunized with 1 μg of MVs per mouse with or without adjuvants at a 3-week interval (30). The mice in the BCG immunization group were intradermally vaccinated with 5×10^5^ CFU of wild-type BCG suspended in 100 μL of sterile PBS. The adjuvants used in this study were 10 µg of poly(I:C) (Sigma−Aldrich, St. Louis, MO, USA), 100 µg of Imject Alum Adjuvant (Thermo Fisher Scientific) and 1 µg of EcN-MVs per mouse. Five weeks after the first immunization, sample collection was performed as previously reported (7, 30, 31). Briefly, saliva was collected after intraperitoneal administration of 0.08 mg/mouse pilocarpine and 0.04 mg/mouse isoproterenol. After saliva collection, the mice were anesthetized via intraperitoneal administration of medetomidine (0.3 mg/kg), midazolam (4 mg/kg) and butorphanol (5 mg/kg) (MMB) and sacrificed via exsanguination. To obtain serum, whole blood was centrifuged for 10 min at 3000×g and 4°C. Additionally, the lungs and nasal cavities were washed with PBS containing 1% bovine serum albumin.

### 2.7 Enzyme-linked immunosorbent assays

The development of humoral immunity was confirmed by ELISAs as described previously, with some modifications (7, 31). Briefly, whole-cell lysates were prepared from BCG cells via bead beating 5 times (5000 rpm for 30 s each time) in carbonate buffer (pH 9.6), followed by centrifugation at 12,000 × g for 20 min at 4°C and passage through 0.45- and 0.22-µm filters. Then, the lysates were diluted to a concentration of 20 μg/mL and pipetted into a 96-well ELISA plate (SUMITOMO BAKELITE, Tokyo, Japan). The BCG lysates were immobilized overnight at 4°C. After the coated antigen was blocked by the addition of 5% skim milk in PBS with 0.05% Tween 20 (PBS-T), the samples collected from the immunized mice (serum, saliva, nasal wash and lung wash) were diluted in 5% skim milk in PBS-T and added to the wells. After overnight incubation at 4°C, diluted horseradish peroxidase-linked secondary antibodies (anti-mouse IgG and anti-mouse IgA antibodies (Cytiva, Wilmington, DE, USA)) were added to the wells at a dilution of 1:5000. The peroxidase substrate SureBlue (SeraCare Life Sciences, Milford, MA, USA) was subsequently added, and the reaction was stopped with 1 M HCl. BCG-specific antibodies were detected via colorimetry, and the absorbance was measured at 450 nm.

### 2.8 BCG challenge

Five weeks after the first immunization, the mice were challenged as previously reported (24). In brief, 5×10^5^ CFU of BCG containing pMV361-Km was suspended in 40 μL of sterile PBS and intranasally administered to the mice under anesthesia with MMB. Two weeks after the challenge, the mice were sacrificed under anesthesia with MMB. The lungs were subsequently homogenized in 1 mL of sterile ultrapure water via bead beating with 28 mm diameter zirconia beads at 5000 rpm for 30 s, and lung cells were lyzed by 0.1% Triton X-100. The lysate was diluted with sterile ultrapure water and plated on Middlebrook 7H10 agar supplemented with oleic acid, bovine serum albumin, dextrose and 10 μg/mL kanamycin. The plates were incubated at 37°C for 20 days, after which the CFUs were counted.

### 2.9 Mass spectrometry

Proteins from two biological replicates of both the s-MVs and p-MVs were identified via MS. Briefly, MV-associated proteins were concentrated by trichloroacetic acid precipitation and separated via sodium dodecyl sulfate–polyacrylamide gel electrophoresis (SDS−PAGE) on a 12% polyacrylamide gel with 10 µg of protein in each lane. The resulting gel was stained with 10% Coomassie Brilliant Blue (G-250) solution, and 4 pieces were excised from each lane. The samples were reduced, alkylated and trypsinized according to an in-gel digestion method (32). The peptides extracted from the gel were subjected to nanoscale liquid chromatography (nanoLC)–MS/MS analysis with a system consisting of an Orbitrap Velos Pro (Thermo Fisher Scientific) coupled with an Advance UHPLC (Brucker, Billerica, MA) and an HTC-PAL autosampler (CTC Analytics, Zwingen, Switzerland). Each peptide sample was separated with both a trap column (5-µm C18 L-column) and an analytical column (3-µm C18 L-column) (Chemicals Evaluation and Research Institute, Tokyo, Japan) (32). The mobile phases were 0.1% formic acid in water (A) and 100% acetonitrile (B). Gradient elution of the peptides was performed from 5–40% B over 40 min and 40–95% B over 1 min, after which a concentration of 95% B was maintained for 9 min; the flow rate was 200 nL/min. Full MS spectra were obtained with an Orbitrap mass spectrometer in the mass/charge (m/z) range of 300–1800 with a resolution of 60,000 at 400 m/z. For automated gain control (AGC), the 12 most intense precursor ions were selected for MS/MS analysis. The raw data were processed using ProteoWizard msConvert (https://proteowizard.sourceforge.io/) and searched against the *M. tuberculosis* H37Rv protein sequence database (the SwissProt_2019_11 database) using the MASCOT algorithm (ver. 2.7.0, Matrix Science Inc., Boston, MA, USA).

Scaffold (version Scaffold_4.11.0, Proteome Software Inc., Portland, OR, USA) was used to validate peptides and proteins identified via MS/MS. We compared the proteins identified from the s-MVs and p-MVs and found those that were uniquely expressed in both types of MV. Gene Ontology (GO) and Kyoto Encyclopedia of Genes and Genomes (KEGG) enrichment analyses were performed using the Database for Annotation, Visualization and Integrated Discovery (DAVID; https://david.ncifcrf.gov/home.jsp).

### 2.10 Western blotting

Proteins in the BCG-derived MVs were extracted in an equal volume of loading buffer containing SDS. Proteins separated by SDS−PAGE were transferred to Immobilon-P PVDF membranes (Millipore), and the membranes were probed with antibodies against lipoarabinomannan (LAM) (clone TB, kindly provided by Otsuka Pharmaceutical Corporation, Tokushima, Japan) and the 19-kDa lipoprotein LpqH (IT-12, BEI Resources).

### 2.11 *In vitro* trained immunity model

THP-1 Mφs were trained as described previously, with some modifications (33, 34). Briefly, differentiated THP-1 Mφs in 24-well plates were incubated with PBS (negative control) or 1 µg/mL s-MVs for 24 h. The cells were washed twice with prewarmed PBS and then incubated for 2 days in culture medium; the medium was changed 6 h before restimulation. The cells were restimulated with PBS, 100 ng/mL lipopolysaccharide (LPS) (35–37), 100 ng/mL Pam3CSK4 (35, 38) or 1 µg/mL s-MVs for 3 h and then washed twice with ice-cold PBS. Finally, total RNA was purified using the Isogen and Direct-zol™ RNA MicroPrep kit.

### 2.12 Statistical analysis

The data obtained from *in vivo* or *in vitro* experiments are presented as the means and standard errors of the means or the means and standard deviations, respectively. Comparisons among more than three groups were performed via one-way analysis of variance followed by Tukey’s HSD test using GraphPad Prism (GraphPad Software, Boston, MA). Comparisons between two groups were performed via unpaired *t* -tests using GraphPad Prism. Differences were considered statistically significant at a value of *P*<0.05.

## 3 Results

### 3.1 Comparison of the BCG-MVs obtained after culture under different conditions

**Figure 1:**
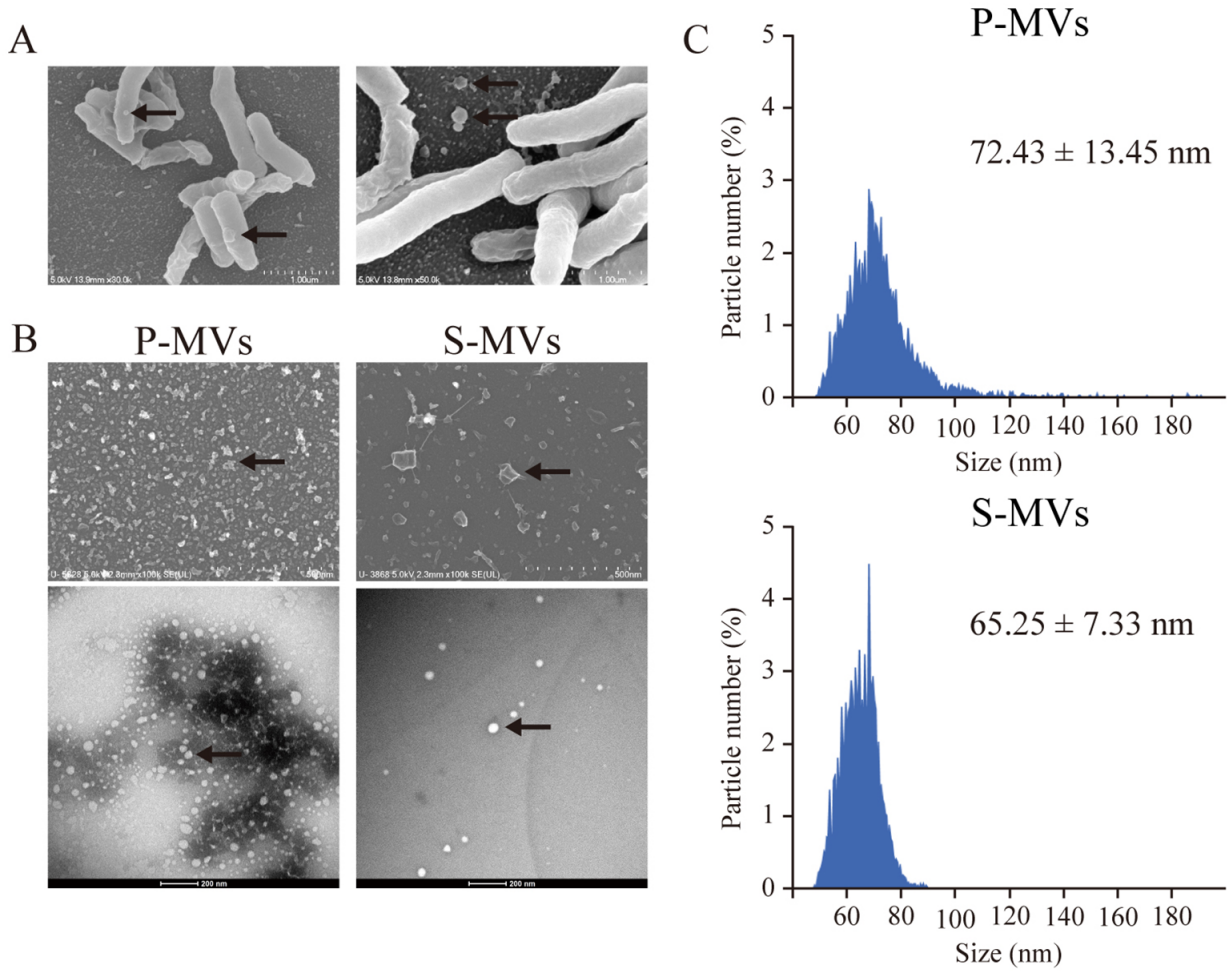
Morphological analysis of membrane vesicles (MVs) from *Mycobacterium bovis* BCG cultured under different conditions. **(A)** Scanning electron microscopy (SEM) revealed MV blebbing on the BCG surface. **(B)** Images of MVs from BCG statically cultured in Sauton’s medium (s-MVs) and BCG planktonically cultured in Muller–Hinton II broth (p-MVs) obtained by field emission SEM (upper) and transmission electron microscopy (lower). **(C)** Analyses of the MV size distribution and particle number via nanoscale flow cytometry.

To investigate whether BCG-MVs could be used as novel TB vaccines, we first aimed to determine the optimal culture conditions for the MV donor bacilli by comparing MVs obtained from statically cultured BCG in nutrient-restricted Sauton’s medium (s-MVs) and those obtained from planktonically cultured BCG in nutrient-rich medium via ultracentrifugation (p-MVs). We first examined whether the morphological and biochemical properties of MVs were affected by the culture conditions of the donor bacilli. SEM analysis revealed that BCG produced MVs by blebbing (**Figure 1A**). We then confirmed the vesicular structure of the purified BCG-MVs by FE-SEM and TEM (**Figure 1B**). EM analysis revealed that smaller MVs (φ 20–40 nm) were enriched in p-MVs than in s-MVs. On the other hand, according to the result of the nanoparticle size analyzer, the mean diameter of the s-MVs (65.25 ± 7.33 nm) was smaller than that of p-MVs (72.43 ± 13.45 nm) (**Figure 1C**). These results showed that differences in the donor cell culture conditions may affect MV morphology.

### 3.2 The protein and lipid compositions of MVs depend on the culture conditions

**Figure 2:**
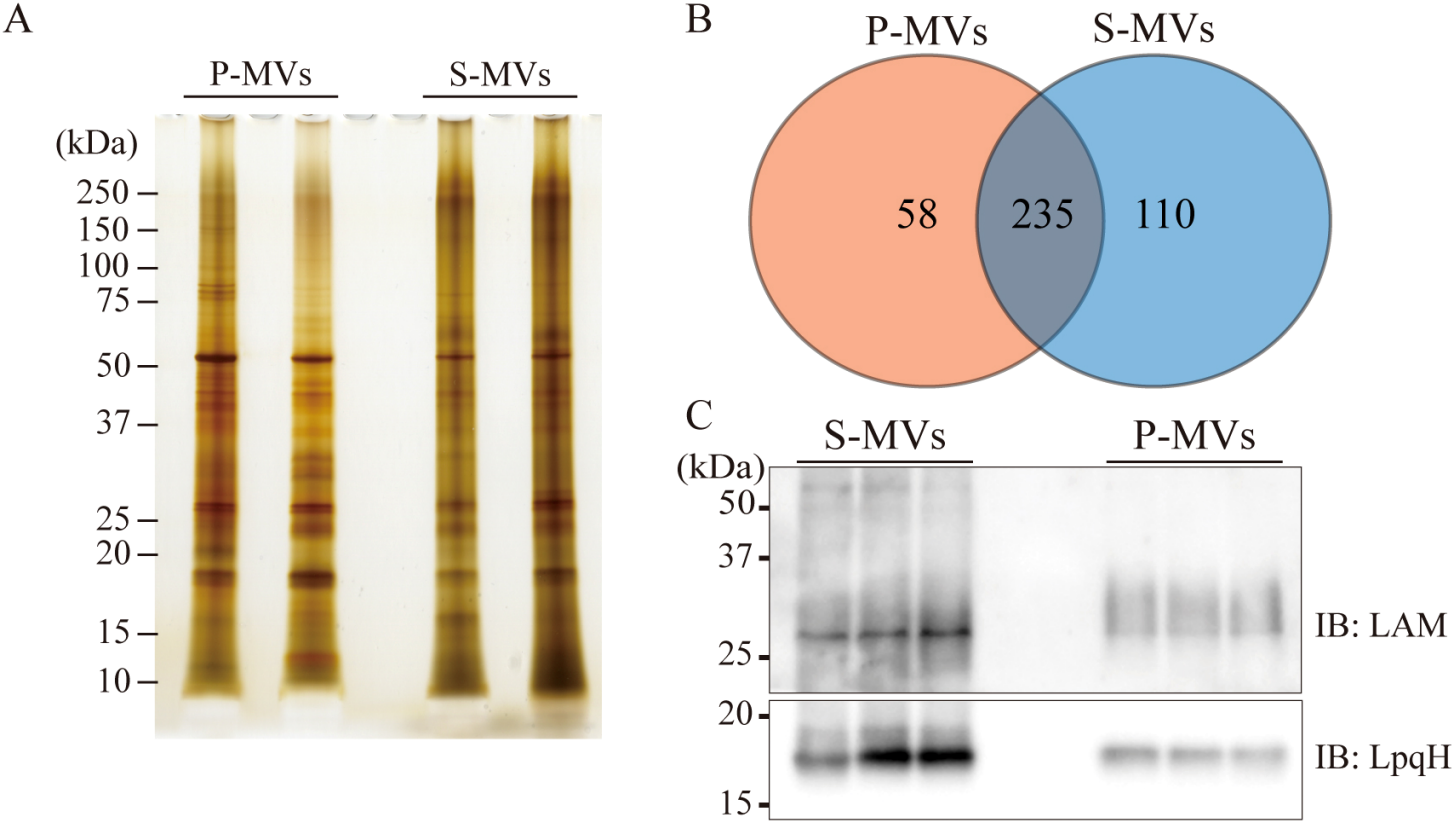
Protein and lipid compositions in BCG-derived membrane vesicles (MVs) obtained from donor bacilli cultured under different conditions. **(A)** MV-associated proteins from static and planktonic culture-derived BCG-MVs (s-MVs and p-MVs, respectively) were separated via SDS−PAGE. **(B)** Comparison of the s-MV and p-MV protein contents with two sets of biological replicates. **(C)** The levels of the glycoproteins lipoarabinomannan (LAM) and 19 kDa lipoprotein (LpqH) in s-MVs and p-MVs were compared via Western blotting with three sets of biological replicates each with three technical replicates.

Because the difference in size distribution between the s-MVs and p-MVs may be caused by the presence of different proteins and lipids in the MVs, we compared the proteins expressed in both types of BCG-MVs (**Figure 2A**). Global LC−MS/MS analysis revealed that 58 proteins were unique to p-MVs and 110 proteins were unique to s-MVs, while 235 proteins were expressed in both types of MVs (**Figure 2B**). Overall, the number of identified proteins was greater in s-MVs than in p-MVs. Moreover, functional annotation clustering via GO and KEGG enrichment analyses revealed that various metabolic pathways (such as valine synthesis, gluconeogenesis, TCA cycle, pyrimidine metabolism and biosynthesis of amino acids) and proteasomal catabolic pathways, which consist of the prokaryotic ubiquitin-like protein (pup)-dependent protein degradation system (39), were enriched in the proteins expressed in the s-MVs (**Supplementary Figures S1A, B**). Next, we compared the amounts of LAM and LpqH, which are components of the mycobacterial cell wall and cell membrane, in the MVs. These molecules are well-known virulence factors in Mtb infection and have immunostimulatory properties that are mediated by Toll-like receptor (TLR) 2 activation (40, 41). The amounts of LAM and LpqH were significantly greater in s-MVs than in p-MVs (**Figure 2C**). These results showed that differences in the donor cell culture conditions may affect the protein and lipid composition in the isolated MVs.

### 3.3 The innate immune response induced by MVs depends on the culture conditions of the MV donor bacteria

**Figure 3:**
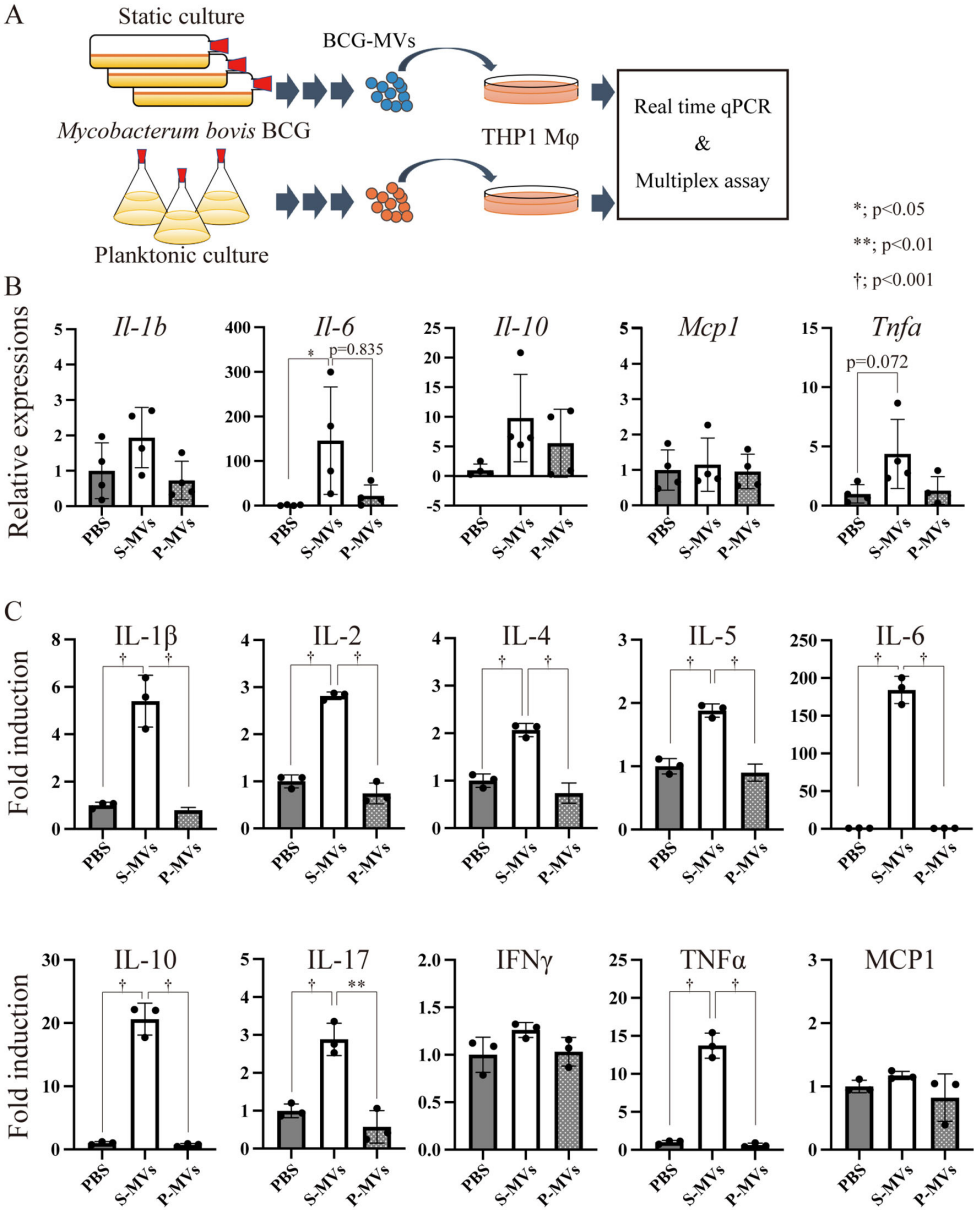
Immunostimulatory properties of BCG-derived membrane vesicles (MVs) in wild-type THP-1 macrophages (Mφs). **(A)** Design of the experimental procedure. THP-1 cells were stimulated with MVs derived from BCG statically cultured in Sauton’s medium (s-MVs) or BCG planktonically cultured in Muller-Hinton II or 7H9-based broth (p-MVs) for 3 h (qPCR, N=4) or 24 h (Bio-Plex assay, N=3). **(B)** The expression levels of inflammation-related genes were evaluated via qPCR with more than three technical replicates. **(C)** Production of cytokines and chemokines by MV-treated THP-1 Mφs was assessed via a Bio-Plex assay. The data are presented as the means and SDs. *P<0.05; **P<0.01; †*P*<0.001 as determined via Tukey’s test.

Next, we examined whether alterations in the molecular composition of MVs caused by the different donor cell culture conditions may affect the immunostimulatory properties of the MVs. Differentiated THP-1 Mφs were stimulated with s-MVs or p-MVs for 3 h (**Figure 3A**), after which the transcription levels of inflammation-related genes were analyzed. Compared with PBS treatment, s-MV treatment significantly increased the expression levels of interleukin (IL)-6. With the exception of monocyte chemotactic protein 1 (MCP1), the levels of IL-1β, IL-10 and tumor necrosis factor alpha (TNFα) tended to increase by s-MV treatment (**Figure 3B**). However, treatment with p-MVs did not affect the expression levels of these genes.

Next, the levels of secreted cytokines were analyzed via multiplex analysis. Treatment with s-MVs increased the production of various cytokines, such as IL-1β, IL-2, IL-4, IL-5, IL-6, IL-10, IL-17 and TNFα, but not interferon gamma or MCP1 (**Figure 3C**). However, p-MVs did not affect the production of these cytokines. The immunostimulatory property of s-MVs depends on the TLR2-mediated signaling pathway because the increased production of cytokines, except MCP1, induced by treatment with s-MVs was abolished in TLR2-knockout THP-1 cells (**Supplementary Figure S2**). These results showed that the culture conditions of the MV donor bacteria affect not only the components of MVs but also their immunostimulatory activities. Moreover, s-MVs strongly stimulate the host innate immune response via TLR2.

### 3.4 Comparison of p-MV and s-MV immunogenicity

**Figure 4:**
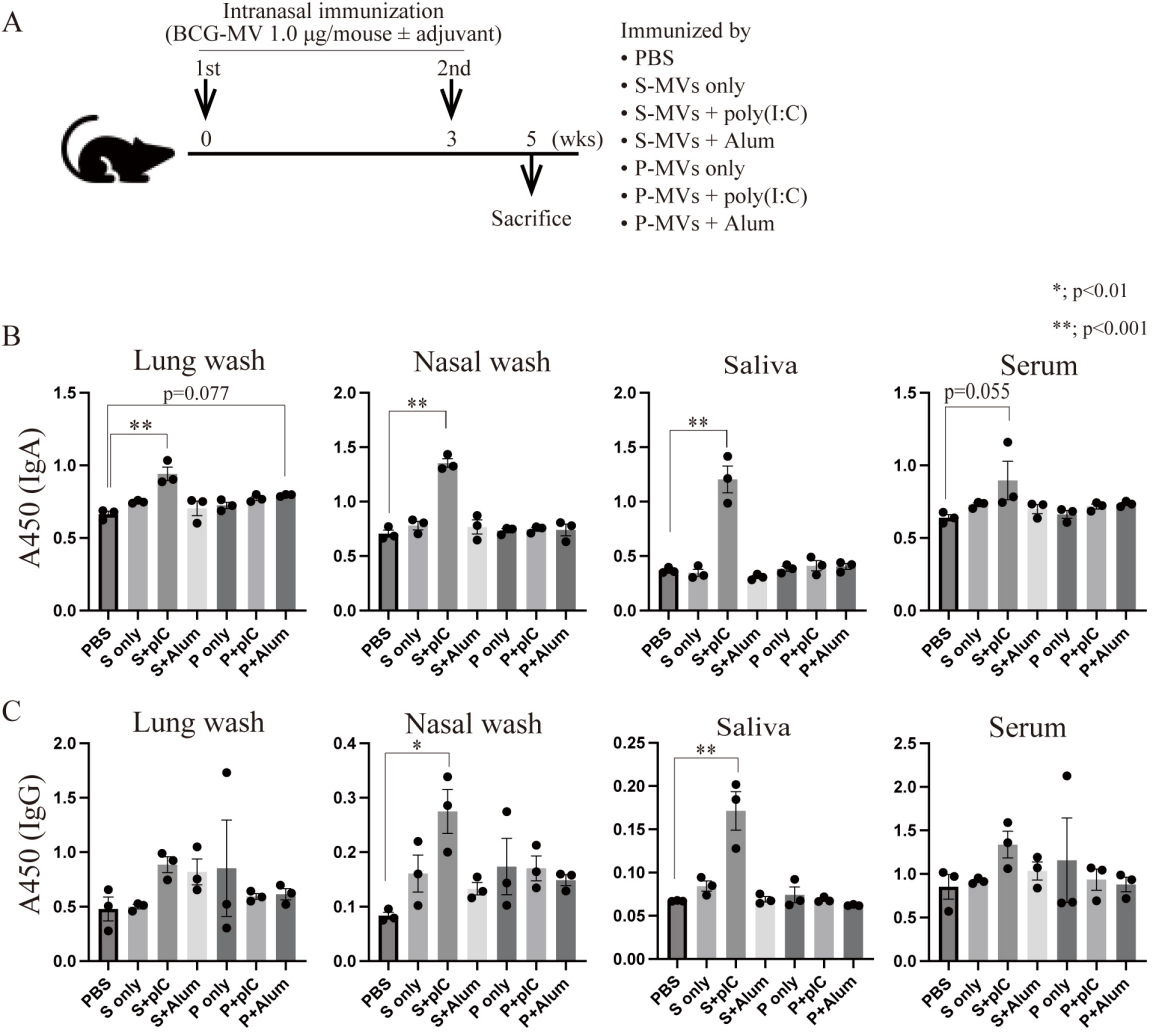
Differences in the immunogenicity of BCG-derived membrane vesicles (MVs) cultured under different conditions. **(A)** Immunization regimen. Six- to eight-week-old female BALB/c mice were intranasally immunized with MVs derived from BCG statically cultured in Sauton’s medium (s-MVs) or BCG planktonically cultured in Muller-Hinton II broth (p-MVs) with or without adjuvants (poly(I:C) or alum) twice at a 3-week interval (N=3). Two weeks after the second immunization, the mice were sacrificed. **(B)** BCG-specific IgA production was examined via ELISA. **(C)** BCG-specific IgG production was examined via ELISA. P, p-MVs; S, s-MVs; pIC, poly(I:C). The data are presented as the means and SEMs (N=3, two technical replicates). *P<0.01; **P<0.001 as determined via Tukey’s test.

Next, we examined whether the culture conditions of the MV donor bacteria affect MV immunogenicity *in vivo*. We intranasally immunized mice twice with s-MVs or p-MVs at a 3-week interval and assessed BCG-specific antibody production (**Figure 4A**). These findings showed that the MVs could not induce the production of BCG-specific antibodies in the absence of an adjuvant. In contrast, significant induction of BCG-specific IgA was observed in the lung wash, nasal wash and saliva samples, but BCG-specific IgA in serum tended to be induced only when the mice were immunized with s-MVs and poly(I:C) but not alum (**Figure 4B**). Moreover, significant induction of BCG-specific IgG was detected in nasal washes and saliva upon coadministration of s-MVs and poly(I:C) (**Figure 4C**). However, p-MVs could not induce antibody production even when adjuvants were coadministered. These results showed that s-MVs stimulated adaptive immune responses more strongly than p-MVs did. Furthermore, s-MVs induced antimycobacterial immunity only when combined with poly(I:C). Poly(I:C), a double-stranded RNA, is an agonist of TLR3 and stimulates the T-helper 1 (Th1)-polarizing immune response by activating NF-κB-regulated gene expression (42). On the other hand, alum usually stimulates the Th2-polarizing immune response (43). Thus, Th1-type adjuvants may be more favorable than Th2-type adjuvants when combined with s-MVs.

### 3.5 Differences in antibody production according to immunization route

**Figure 5:**
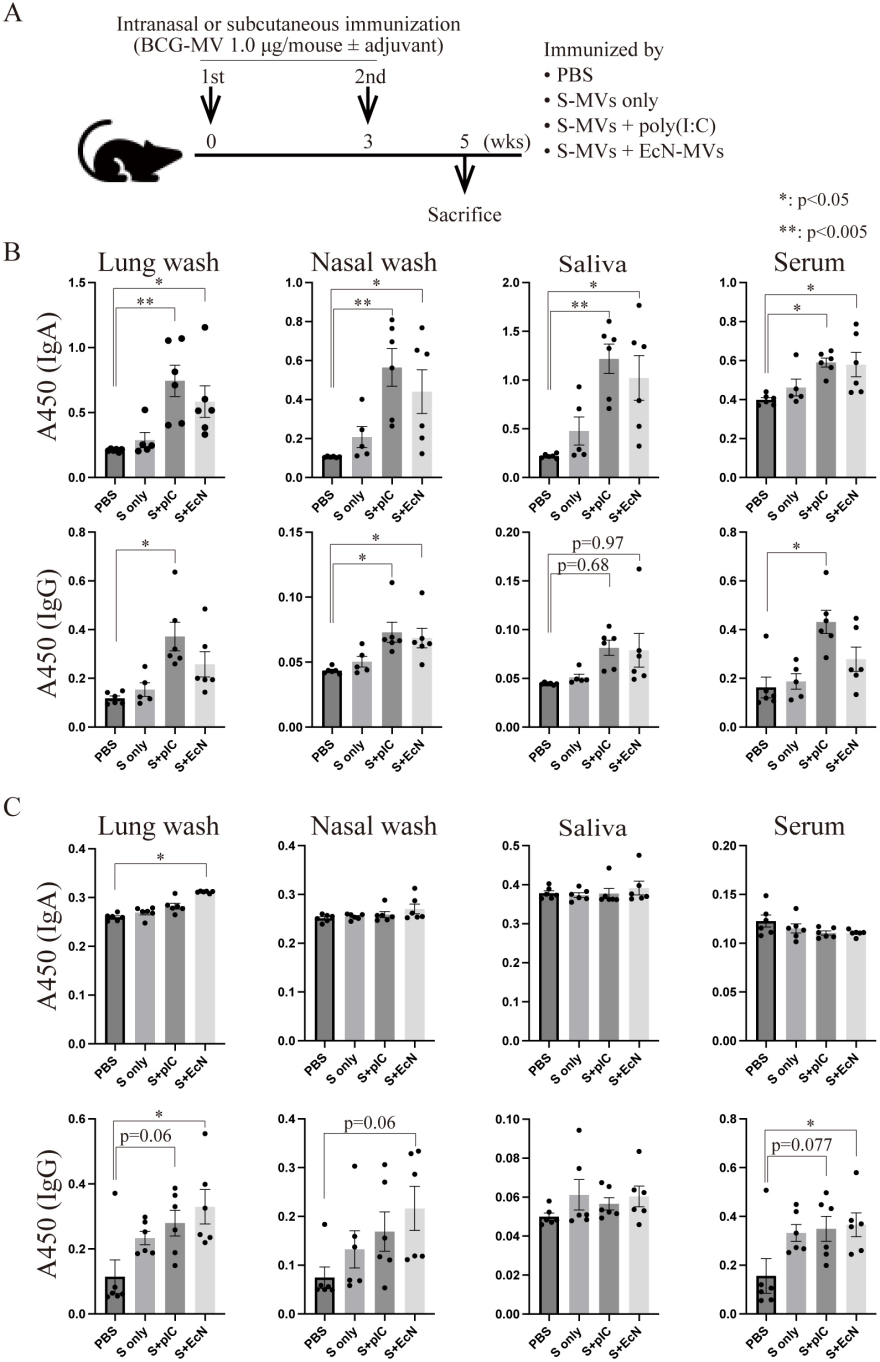
Differences in the induction of antibody production on the basis of the immunization route using membrane vesicles derived from BCG statically cultured in Sauton’s medium (s-MVs). **(A)** Immunization regimen. Six- to eight-week-old female BALB/c mice were intranasally or subcutaneously immunized with s-MVs with or without adjuvants (poly(I:C)- or flagellar-deficient *Escherichia coli* Nissle 1917 (EcN)-derived MVs) twice at a 3-week interval (N=5-6). Two weeks after the second immunization, the mice were sacrificed. **(B)** BCG-specific IgA and IgG antibody production after intranasal immunization was examined via ELISA. **(C)** BCG-specific IgA and IgG antibody production after subcutaneous immunization was examined via ELISA. S, s-MVs; S + pIC, s-MVs with poly(I:C); S + EcN, s-MVs with EcN-MVs. The data are presented as the means and SEMs (N=5-6, two technical replicates). *P<0.05; **P<0.005 as determined via Tukey’s test.

A previous study revealed that EcN-MVs, another Th1-type adjuvant (44), have greater adjuvanticity than poly(I:C) (26). We next investigated whether poly(I:C) or EcN-MVs are more suitable adjuvants for s-MVs (**Figure 5A**). First, BCG-specific antibody production in intranasally immunized mice was assessed. Compared with the control, both the poly(I:C) and EcN-MV adjuvants significantly induced the production of BCG-specific IgA, as detected in the lung washes, nasal washes, saliva and serum (**Figure 5B**). However, with respect to adjuvanticity for IgG production, poly(I:C) tended to be superior to EcN-MVs. TB-specific secretory IgA production is important for TB prevention, whereas TB-specific IgG enhances TB infection (45). However, IgG is not necessarily a harmful factor for TB (46, 47). We also compared the adjuvanticities of poly(I:C) and EcN-MVs in subcutaneously immunized mice (**Figure 5A**). Unlike intranasal immunization, the adjuvanticity of EcN-MVs tended to be superior to that of poly(I:C) upon subcutaneous immunization (**Figure 5C**). MVs have both high adjuvanticity and high immunogenicity; however, s-MVs alone did not induce significant production of mycobacterial antibodies upon either intranasal or subcutaneous immunization (**Figures 4B, C**). These data suggest that EcN-MVs are needed to enhance the mycobacterial humoral immune response induced by subcutaneous immunization with s-MVs.

### 3.6 Immunization with BCG-MVs confers protection against mycobacterial infection

**Figure 6:**
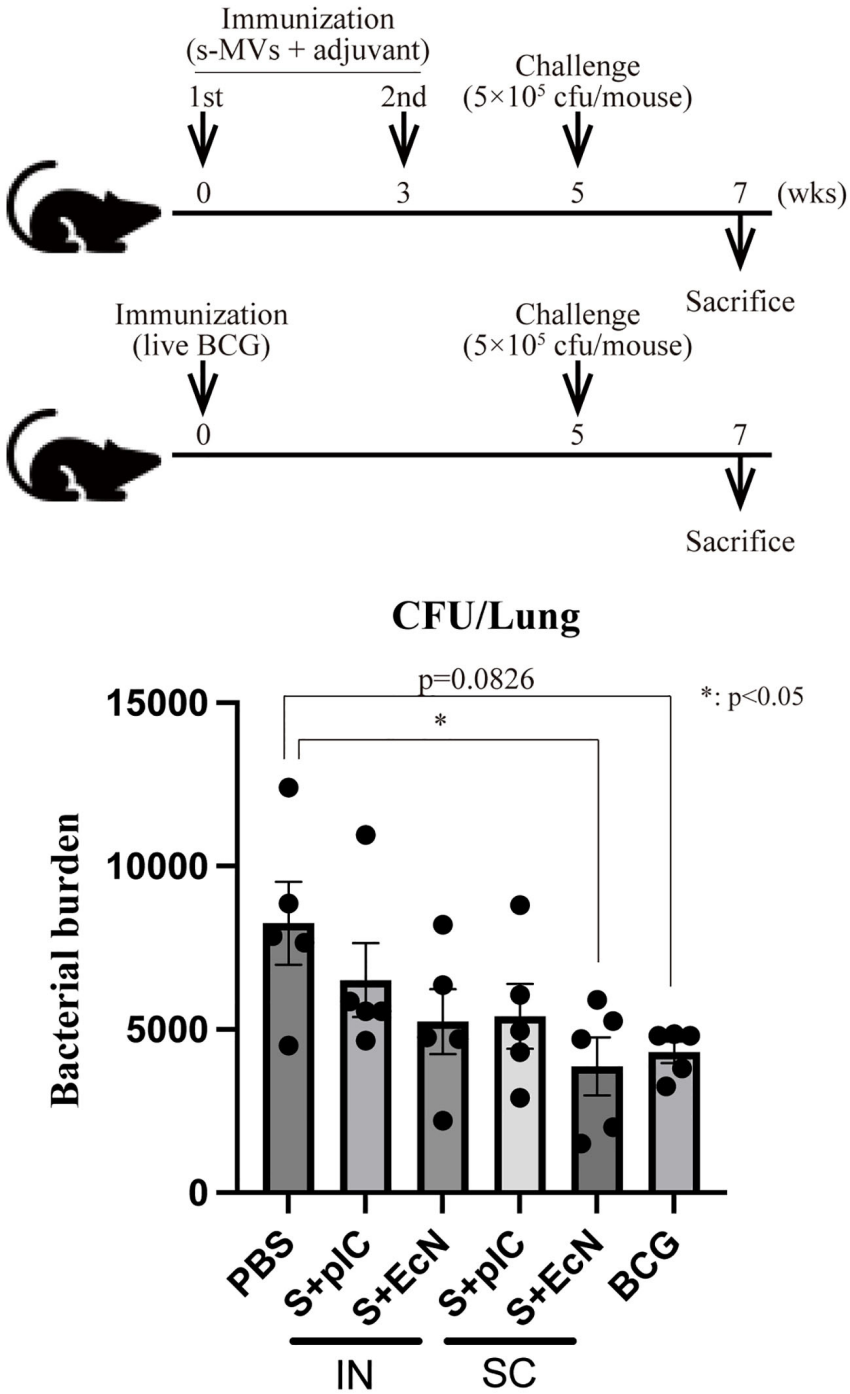
Induction of tuberculosis immunity by membrane vesicles derived from BCG statically cultured in Sauton’s medium (s-MVs). The immunization regimen and protocol are shown in the upper panel. Six- to eight-week-old female BALB/c mice were intranasally (IN) or subcutaneously (SC) immunized with s-MVs with or without adjuvants (poly(I:C)- or flagellar-deficient *Escherichia coli* Nissle 1917 (EcN)-derived MVs) twice at a 3-week interval (N=5). The control mice were intradermally immunized with live BCG (5 × 10^5^ CFU/mouse). Five weeks after the first immunization, the mice were infected with 5 × 10^5^ CFU of BCG::pMV261-Km (N=5, two technical replicates). Two weeks after infection, the mice were sacrificed, and CFUs of Km-resistant BCG in the lungs were counted. S + pIC, s-MVs with poly(I:C); S + EcN, s-MVs with EcN-MVs. The data are presented as the means and SEMs. *P<0.05 as determined via Tukey’s test.

Next, we investigated whether immunization with s-MVs confers efficient protection against mycobacterial infection in a BCG challenge study. Mice that were intranasally or subcutaneously immunized with s-MVs with or without poly(I:C) or EcN-MV as an adjuvant twice at a 3-week interval were intranasally challenged with live Km-resistant BCG (5 × 10^5^ CFU/mouse) at 5 weeks after the first immunization. Moreover, mice intradermally immunized with live BCG were also challenged with live Km-resistant BCG at 5 weeks after immunization. At 2 weeks after challenge, intranasal immunization with s-MVs did not significantly reduce the bacterial burden in the mouse lungs (**Figure 6**) despite inducing BCG-specific antibody production (**Figure 5B**). Among the groups receiving subcutaneous immunization, the combination of s-MVs and EcN-MVs significantly reduced the bacterial burden in the lungs to a level comparable to that in the mice intradermally immunized with BCG (**Figure 6**).

### 3.7 s-MV induction of trained immunity *in vitro*

**Figure 7:**
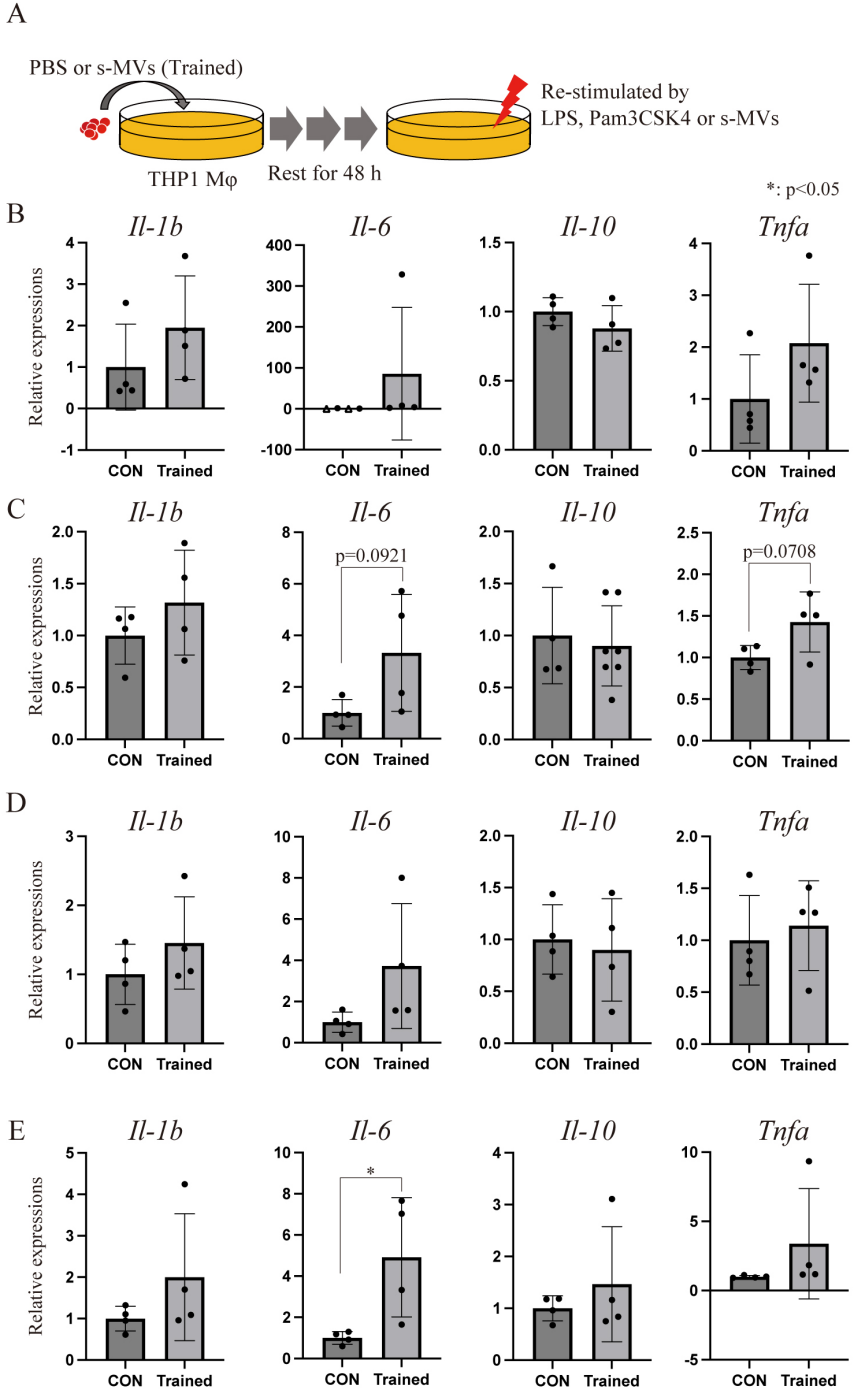
Membrane vesicle (MV)-induced trained immunity *in vitro*. **(A)** Experimental procedure by which THP-1 macrophages (Mφs) were trained using membrane vesicles from BCG statically cultured in Sauton’s medium (s-MVs). THP-1 Mφs were trained with st-MVs or treated with PBS (untrained) for 24 h and then incubated for an additional 48 (h) **(B)** Basal transcription levels of inflammation-related genes in trained and untrained (CON) THP-1 Mφs treated with PBS for 3 (h) Transcription levels of inflammation-related genes in trained and untrained THP-1 Mφs upon restimulation with s-MVs **(C)**, Pam3CSK4 **(D)** or lipopolysaccharide (LPS) **(E)** for 3 (h) The data are presented as the means and SDs (N=4, more than two technical replicates). *P<0.05 as determined via Tukey’s test. The open triangles (△) indicate samples in which gene expression could not be detected.

The BCG vaccine confers not only TB immunity but also trained immunity (17, 18, 33). A recent study revealed that MVs from *E. coli* induce trained immunity in mice (16). We therefore investigated whether s-MVs could induce trained immunity *in vitro*. We trained THP-1 Mφs with BCG-MVs, and the trained cells were restimulated with the TLR4 agonist LPS, the TLR2 agonist Pam3CSK4, or s-MVs followed by 48 h of rest (**Figure 7A**). In the absence of restimulation (PBS treatment), the basal expression levels of IL-6, IL-1β and TNFα in THP-1 Mφs tended to increase with training (**Figure 7B**). The expression level of IL-6 in trained cells also tended to increase when the cells were restimulated with Pam3CSK4 and s-MVs (**Figures 7C, D**, respectively). Moreover, the expression level of IL-6 in trained cells significantly increased when the cells were restimulated with LPS (**Figure 7E**). These data may indicate that vaccination with s-MVs confers not only specific protection against TB infection but also broad protection against gram-negative pathogen infection in a TLR4-dependent manner.

## 4 Discussion

In the present study, we investigated whether BCG-MVs could be used as a novel TB vaccine. Upon comparison of two different BCG-MV preparations, we found that s-MVs, obtained from static-cultured BCG in nutrient-restricted Sauton’s medium, but not p-MVs obtained from planktonic-cultured BCG in nutrient-rich medium, induced BCG-specific immunity and reduced the bacterial burden in mice. In addition, we showed that s-MVs induced trained immunity. In this study, we demonstrated that developing a novel TB vaccine using s-MVs is possible, and such a vaccine may also protect against other infectious diseases.

Our data revealed that s-MVs, but not p-MVs, contained increased levels of LAM and LpqH and strongly induced inflammation in a TLR2-dependent manner. The components of MVs are affected by the milieu of the MV donor bacilli, including both their nutrient status and culture methodology (planktonic or static) (25, 48). Baena et al. reported that the difference in the components of 7H9-based medium and Sauton’s medium affects the expression of various metabolism-related genes in Mtb and that cultivation in Sauton’s medium increases lipid and lipoprotein synthesis-related gene expression (49). These findings support our observations that MVs from BCG cultured in nutrient-restricted Sauton’s medium may contain more lipids and lipoproteins than MVs from BCG cultured in nutrient-rich medium. On the other hand, Takahara et al. reported that *Pseudomonas aeruginosa*-derived MVs from static culture contain more lipids than MVs obtained after planktonic culture, which results in greater immunostimulatory properties (48). We expect that differences in both nutritional status and culture methodology (planktonic or static) affect bacterial metabolism, resulting in BCG-MVs with different immunostimulatory properties.

MS analysis of the MV-associated proteins led to the identification of more proteins in s-MVs than in p-MVs (345 versus 293 proteins, respectively). The subsequent enrichment analysis suggested that various metabolic pathways and pup-dependent proteasome catabolic pathways are activated in the s-MV donor bacilli compared with the p-MV donor bacilli. Nutrient restriction activates lipid biosynthesis (49, 50) and the pup-proteasome system (39) in mycobacteria. Additionally, in eukaryotes, the activity of the ubiquitin−proteasome system affects lipid biosynthesis (51). Activation of the pup-proteasome system in s-MV donor bacilli may result in increased amounts of the immunostimulatory factors LAM and LpqH.

The increased number of proteins identified in s-MVs may also lead to the increased immunogenicity of s-MVs compared with p-MVs, and s-MVs may contain more protective vaccine antigens than p-MVs do. Indeed, known protective vaccine antigens against TB Ag85A, MPT64, HspX and GroEL2 was abundant in s-MVs, as determined via LC−MS/MS (**Supplementary Table S1**) (52–55). However, which antigens in the s-MVs effectively induce TB immunity remains unclear. Thus, further study is needed to identify the protective antigens.

TLR agonists, including TLR2 agonists, are a major category of vaccine adjuvants (42, 56). Although s-MVs exhibited greater immunostimulatory effects through TLR2 than p-MVs did, neither intranasal nor subcutaneous administration of s-MVs alone could effective BCG-specific immunity. There are three possible reasons for this result. First, the agonistic activity of s-MVs against TLR2 may be weaker than that of other TLR2 adjuvants and may be inadequate to induce antibody production. Second, immunization was performed only twice in this study. Three or more immunizations could have induced effective TB-specific immunity. Third, the immunoinhibitory properties of mycobacterial MVs may interfere with the immunogenicity of BCG-MVs (13, 57).

Recent clinical trial data have shown that intradermal revaccination with BCG is effective for preventing Mtb infection (58, 59). However, the BCG vaccine sometimes causes adverse events, among which skin abscess at the BCG injection site are the most common, and severe disseminated BCG infection rarely occurs (3, 6). The BRACE study revealed that the risk of adverse events upon revaccination, such as abscesses at the injection site and regional lymphadenopathy, increased despite the exclusion of individuals who experienced adverse BCG infection in initial BCG vaccination (60, 61). Theoretically, the frequency of severe adverse infections associated with BCG revaccination may be similar to or greater than that associated with initial BCG vaccination, especially in immunocompromised individuals. Recent studies showed that intravenous BCG vaccination confers stronger protection against Mtb infection in an experimental macaque TB model than classical intradermal vaccination does (4, 5). However, it remains unclear whether intravenous BCG vaccination confers protection against TB in people of all age groups and is applicable for human use. With respect to these disadvantages, safe vaccines that can be used for both initial vaccination and revaccination and replace the BCG vaccine need to be developed (62, 63). We expect MV-based TB vaccines to be applicable to a safe alternative vaccine for the initial BCG vaccination for immunocompromised people and a booster vaccine for people of all age groups who experienced the initial BCG vaccination. Further investigations are needed to clarify how long MV-induced TB immunity continues compared with BCG vaccine, and whether s-MVs can be applied as a booster vaccine to a previously administered BCG vaccine.

Recent studies have shown that the BCG vaccine confers not only TB immunity but also protection against infectious diseases other than TB (18). The BCG vaccine alters the gene expression signatures and phenotypes of innate immune cells, especially HSPCs in bone marrow, through epigenetic reprogramming (17, 34). However, it remains unclear why BCG induces trained immunity in bone marrow cells, which is far from the site of inoculation. Nanoparticles, such as exosomes and MVs, can spread throughout the entire body through the blood and lymphatic system. Our data revealed that the steady-state expression of IL-6 in THP-1 Mφs tended to increase in the BCG-MV-trained group. In addition, LPS stimulation increased IL-6 production to a greater extent in the BCG-MV-trained group than in the control group *in vitro*. Our data suggest that BCG-MVs may play a key role in the induction of trained immunity by the BCG vaccine.

Finally, in this study, mycobacterial immunity induced by s-MVs was confirmed upon infection with BCG, not Mtb. Although BCG is quite similar to Mtb, further investigations are needed to confirm the effectiveness of s-MV vaccines.

## 5 Conclusions

In this study, we showed that BCG-derived MVs are novel TB vaccine candidates. We also found that the immunogenicity of MVs depends on the culture conditions of the donor bacilli and that s-MVs produced by BCG statically cultured in nutrient-restricted medium are more immunogenic than MVs produced by BCG planktonically cultured in nutrient-rich medium. Furthermore, s-MVs activate innate immune memory, also known as trained immunity. Vaccines based on s-MVs may protect against not only TB but also other infectious diseases, such as those caused by gram-negative pathogens, in a TLR4-dependent manner. This study may provide a rationale for developing novel acellular TB vaccines using BCG-MVs.

## Data Availability

The original contributions presented in the study are included in the article/**Supplementary Material**, further inquiries can be directed to the corresponding author/s.

## Supplementary material

Supplementary Figure 1 Functional annotation of the proteins identified in BCG-derived membrane vesicles. **(A)** Gene Ontology enrichment analysis and **(B)** Kyoto Encyclopedia of Genes and Genomes pathway annotation. Related to **Figure 2**.

Supplementary Figure 2 Immunostimulatory properties of BCG-derived membrane vesicles (MVs) in Toll-like receptor 2-deficient THP-1 macrophages. The data are presented as the means and SDs (N=3). **P*<0.05 as determined via Tukey’s test. Related to **Figure 3**.
