# Supplementary figures and images for: BCG-derived acellular membrane vesicles elicit antimycobacterial immunity and innate immune memory
Source: Front Immunol. 2025 Mar 12;16:1534615. doi: 10.3389/fimmu.2025.1534615 (PMC11937015; doi:10.3389/fimmu.2025.1534615)

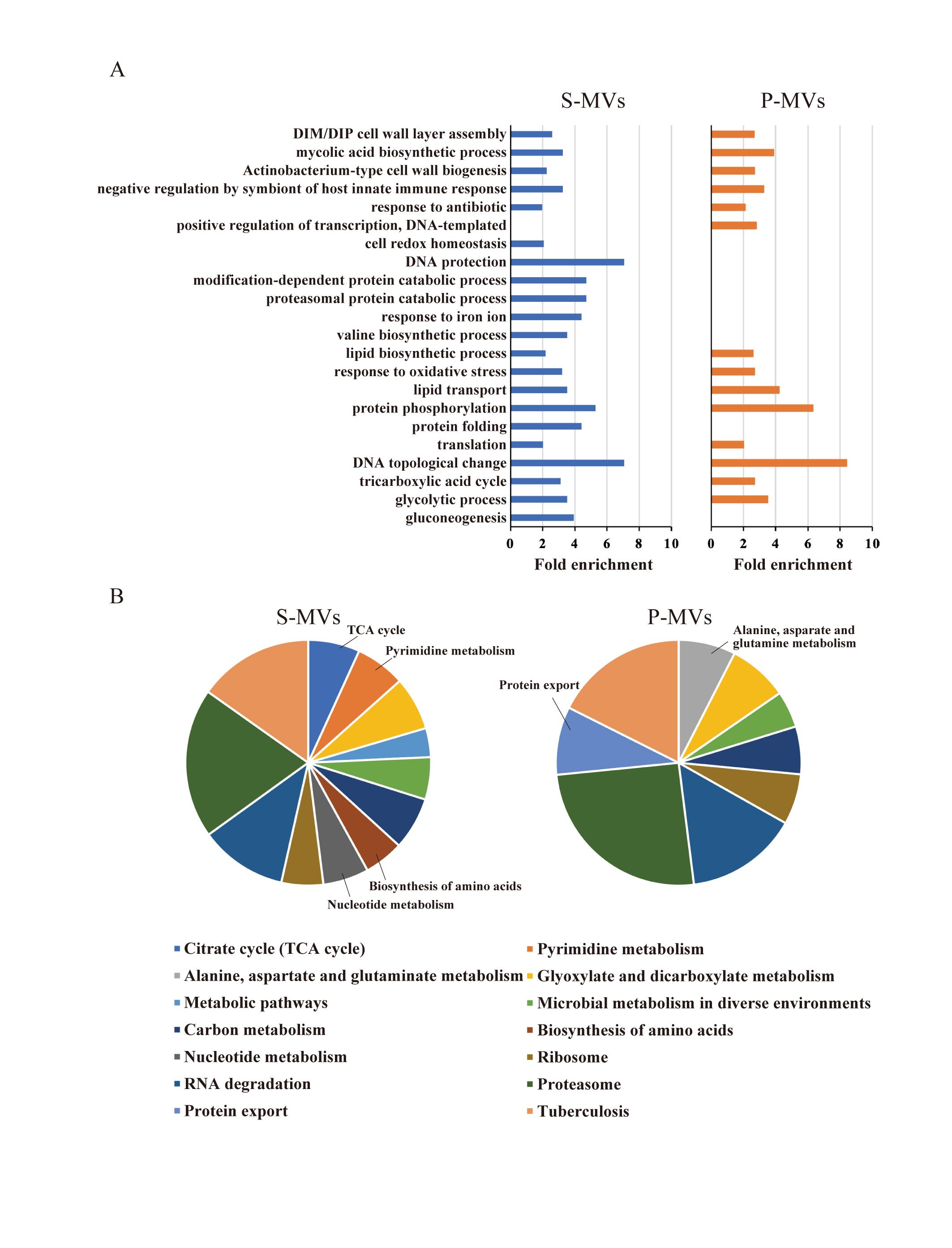

Supplement: Supplementary Figure 1 — Functional annotation of the proteins identified in BCG-derived membrane vesicles. (A) Gene Ontology enrichment analysis and (B) Kyoto Encyclopedia of Genes and Genomes pathway annotation. Related to Figure 2 . [file Image1.jpeg]

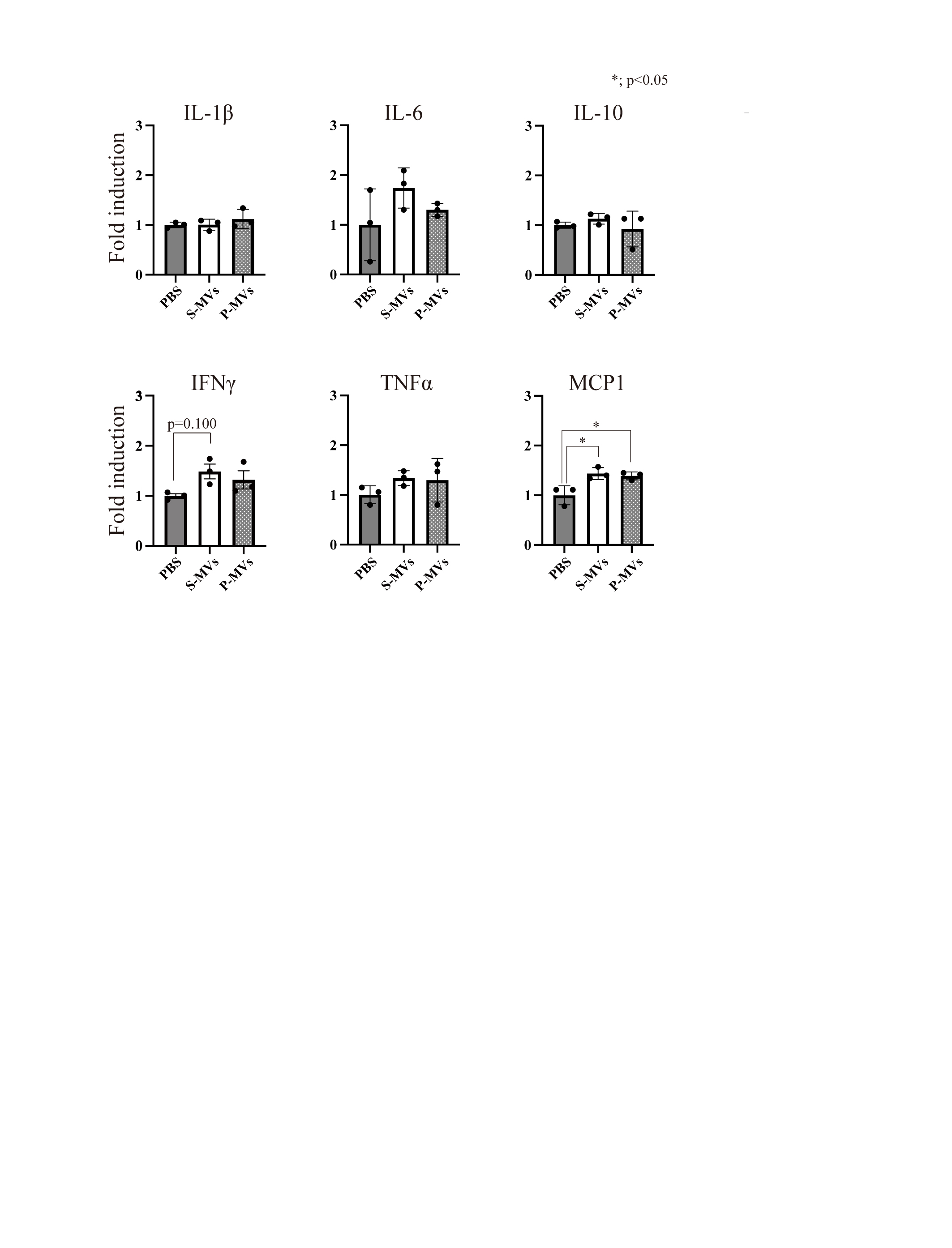

Supplement: Supplementary Figure 2 — Immunostimulatory properties of BCG-derived membrane vesicles (MVs) in Toll-like receptor 2-deficient THP-1 macrophages. The data are presented as the means and SDs (N=3). *P<0.05 as determined via Tukey’s test. Related to Figure 3 . [file Image2.jpeg]
